# AGE MATTERS IN FINANCIAL STRAIN AND SUBJECTIVE HEALTH AND WELL-BEING FOR PEOPLE WITH LTSS NEEDS IN CALIFORNIA

**DOI:** 10.1093/geroni/igad104.2086

**Published:** 2023-12-21

**Authors:** Lei Chen

**Affiliations:** UCSF Philip R. Lee Institute for Health Policy Study, San Francisco, California, United States

## Abstract

Long-Term Services and Supports (LTSS) are a big concern for older adults and people with disabilities. It is important to reset aging and disability silos and create coordinated LTSS networks for people with disabilities of all ages, including those with cognitive impairments, difficulties performing activities of daily living (ADLs) and/or instrumental activities of daily living (IADLs). This study examined how age differentiates the associations among disability status, financial strain, and subjective health and well-being for people with LTSS needs in California. This study used the first cycle of data (2019-2020) from the California Long-Term Services and Supports (CA-LTSS) survey, merged with select data from the California Health Interview Survey (CHIS) (N = 2,030). Multivariate Regressions and Conditional Process Analysis (CPA) were applied to test the hypothesized relationships. Findings show that young and middle-aged participants with LTSS needs struggled to make financial ends meet. Middle-aged and older participants with LTSS needs reported worse self-rated health, and young participants with LTSS needs had severer psychological distress. Participants with difficulty in IADLs or more types of disability difficulties tended to have more financial strain, and these associations were significantly stronger for young participants than older participants. Similarly, participants with difficulty in IADLs or more types of disability difficulties tended to report severer psychological distress, and these associations were significantly stronger for young participants than older participants. The findings suggest the value of approaching younger people with disabilities and target policies and service programs to meet their financial and mental health needs.

